# Evaluating the Effectiveness of Enoxaparin in Treating Pediatric Arterial Thrombosis in Saudi Arabia

**DOI:** 10.3390/children11091139

**Published:** 2024-09-19

**Authors:** Meshary Al-Meshary, Abdulrahman Alotaibi, Nouf S. Alsagri, Asmaa AlZhrani, Husam I. Ardah, Mohammed A. Alnuhait

**Affiliations:** 1 Pharmaceutical Care Department, King Abdulaziz Medical City, National Guard Health Affairs, Riyadh 11426, Saudi Arabia; almesharyme@ngha.med.sa (M.A.-M.); alotaibiab8@ngha.med.sa (A.A.); alsagrino@mngha.med.sa (N.S.A.); alzhranias@ngha.med.sa (A.A.); 2 King Abdullah International Medical Research Center, Riyadh 11481, Saudi Arabia; ardahhu@ngha.med.sa; 3 Pharmaceutical Practices Department, College of Pharmacy, Umm Al-Qura University, Makkah 24381, Saudi Arabia

**Keywords:** pediatric, arterial thrombosis, enoxaparin

## Abstract

Background: Thrombosis is the abnormal formation of blood clots within blood vessels; it results from an imbalance between fibrinolytic, pro-coagulant, and anticoagulant systems. Pediatric arterial thrombosis, especially related to catheter usage, is an emerging issue with limited evidence. This study evaluates the efficacy of enoxaparin in treating arterial thrombosis in pediatric patients at a single center. Methods: A retrospective single-center study included children under 14 years old diagnosed with catheter-related arterial thrombosis (CAT) and treated with low-molecular-weight heparin (LMWH) at King Abdulaziz Medical City between 2016 and 2021. Patients without follow-up at our institution or those using other anticoagulants were excluded. Data collected included age, sex, weight, catheter type, location and degree of thrombosis, ultrasonographic results, treatment duration, hemoglobin and platelet levels, and missed refills. Radiologic confirmation of CAT was required for inclusion. Results: This study included 111 children treated with enoxaparin for non-cerebral arterial thrombosis. The median age at diagnosis was 3 months, with 58% being male patients. Most cases (87%) involved cardiac catheterization, and all were confirmed using ultrasonography. Complete thrombus resolution was achieved in 90% of patients, partial resolution in 8.1%, and 1.8% had no resolution. The median duration of enoxaparin therapy was 20 days. Multivariate analysis indicated that higher age and lower body weight were associated with a higher risk of non-resolution. Indwelling catheters also posed a greater risk of non-resolution compared to cardiac catheters. Conclusions: Enoxaparin proved effective in treating catheter-related arterial thrombosis in children, with high resolution rates and few side effects. This study helps inform treatment strategies in pediatric thrombosis management and highlights the need for further research to refine treatment durations and address patient risk factors.

## 1. Introduction

Thrombosis is the abnormal formation of a blood clot within a blood vessel. It happens because of an imbalance between the body’s fibrinolytic, pro-coagulant, and anticoagulant systems [1]. Adults and children under 1 year of age are the most at risk, with thrombosis causing a 33% mortality rate in infants [1,2]. Arterial thrombi are platelet rich and form at sites of arterial rupture, often referred to as “white thrombi”. In contrast, venous thrombi form in veins where the wall remains intact and are rich in red blood cells, hence they are called “red thrombi” [3]. In adults, arterial thrombosis is often due to atherosclerosis. However, in children, it usually occurs in normal arteries because of the inflammation caused by arterial catheters [4]. Pediatric arterial thrombosis is increasingly recognized as a significant issue, though evidence is limited. It is mostly linked to ischemic stroke or the presence of an arterial catheter [4]. The prevalence of arterial thrombosis in children is thought to be similar to venous thrombosis, with a rate of 8.5 per 10,000 hospital admissions [5]. Pediatric arterial thrombosis can be categorized into catheter-related thrombosis (CAT) and non-catheter-related thrombosis (NCAT) [4]. CAT often results from the use of peripheral indwelling arterial catheters, including those in the extremities (“EIC” in ICU), or during procedures such as blood pressure monitoring or the use of umbilical catheters in neonates and cardiac catheterization [4]. The general incidence of CAT is around 21%, with the highest rates seen in indwelling arterial catheters (ranging from 3.4% to 63%), approximately 20% for umbilical catheters, and 11% for cardiac catheterizations [6]. NCAT is less common and usually results from acquired disorders like Kawasaki disease or congenital conditions such as metabolic abnormalities [4]. Non-cerebral arterial thrombosis in pediatric patients is mostly associated with arterial catheterization and is less frequently reported compared to venous thrombosis (5). Arterial CAT has an incidence of 18%, which includes both partial and complete occlusions, and carries a mortality rate of around 3% [6]. Complications from arterial thrombosis can include limb ischemia or necrosis, differences in leg length, claudication, and loss of future access sites [7]. Due to a lack of evidence-based guidelines, current practices are largely based on expert opinion. Unlike in adults, there are no specific recommendations for the optimal treatment choice or duration in children [5,8]. The Chest Guidelines suggest using low-molecular-weight heparin (LMWH) or unfractionated heparin (UFH) for 5–7 days, with the duration potentially being shorter or longer, and consider thrombolysis or surgical thrombectomy. These recommendations are drawn from limited single-center case studies [6]. Enoxaparin is preferred in children over unfractionated heparin because it requires minimal monitoring, has predictable pharmacokinetics, is easier to administer, and has fewer side effects [8]. Thrombolytics are generally not preferred in children due to the increased risk of bleeding and are reserved only for large arterial thromboses that threaten limb or organ viability [8]. Percutaneous endovascular interventions are a type of microsurgery aimed at restoring blood flow without causing major bleeding. These procedures use radiologic imaging to guide a catheter into the affected vessels. They have a success rate of over 85% and a bleeding rate of 0–10%, but they are often impractical because 50% of patients may require additional angioplasty or thrombectomy. Additionally, these procedures are not feasible for neonates and small children due to the size of the catheters. Open surgical thrombectomy, which provides direct access to the vessel, carries more complications and should be reserved for life-threatening thrombosis [4]. Given the lack of concrete evidence for CAT pharmacological treatment and the appropriate treatment duration in pediatric patients, our retrospective study aims to identify the efficacy of enoxaparin and determine the optimal treatment duration in our population. This will help guide clinicians in selecting the best treatment strategies.

## 2. Method

This study retrospectively included children under the age of 14 who were diagnosed with catheter-related arterial thrombosis (CAT) and treated with low-molecular-weight heparin (LMWH) at King Abdulaziz Medical City between 2016 and 2021. These patients were identified based on diagnosis coding and were subsequently followed up at our institution. Patients were excluded from the study for the following reasons: no follow-up at our institution or follow-up longer than 3 months, use of other anticoagulants, lack of follow-up data due to death (unrelated to CAT or anticoagulation), absence of radiological confirmation of thrombosis, presence of both deep vein thrombosis (DVT) and CAT, and other unspecified reasons. King Abdulaziz Medical City, a tertiary care facility, includes the King Abdullah Specialist Children Hospital, one of the largest pediatric hospitals in the Middle East. The hospital provides care for preterm and term newborns, as well as children with various medical, traumatic, and surgical conditions; it has a capacity of 600 beds. 

### 2.1. Data Collection

The electronic clinical records of all included patients were reviewed to collect the following data: age at diagnosis of CAT, sex, weight, type of catheter (indwelling vs. cardiac), location of CAT and degree of occlusion, results of ultrasonographic examination, treatment duration (starting from the first therapeutic level for UFH or LMWH), hemoglobin and platelet levels from the start of treatment to the end, and the number of missed refills.

### 2.2. Diagnosis and Treatment of CAT

Radiologic analysis proved every clinically suspected case of CAT. A cold and pallid limb, a threatening limb, and the loss of a detectable pulse are some of the clinical indications of CAT. In individuals exhibiting clinical indications of CAT, Doppler ultrasonography is typically used to make the radiologic diagnosis. Ultrasonographic screening is not standard clinical practice at our institution for all patients with an indwelling arterial catheter or after cardiac catheterization. At our institution, children with CAT are usually treated with LMWH or UFH until clinical and radiologic resolution of thrombosis occurs (depend on the treating physician), with LMWH being the preferred agent for outpatients.

### 2.3. Statistical Methodology

R Studio software (version 2022.02.3, The R Foundation for Statistical Computing, Boston, MA, USA) was used for data analysis. The normality of data distribution was checked using the Kolmogorov–Smirnov test, histograms, and Q-Q plots. Continuous variables were presented as median (25th percentile, 74th percentile), and were not normally distributed. Categorical variables were presented as frequencies (%). Multivariate ordinal logistic regression analysis was performed to identify factors associated with various degrees of thrombus resolution (no resolution, partial resolution, or complete resolution), and the strength of associations was presented as an odds ratio (OR) with corresponding 95% confidence intervals (CIs). The cumulative resolution rate was estimated and plotted against the time to resolution. The R package “gtsummary” was used to create summary tables. The “survival”, “ggsurvfit”, and “tidycmprsk” packages were used to generate cumulative incidence curves and associated risk tables. The “VGAM” R package (version 1.1-8) was used to carry out the multivariate ordinal logistic regression analysis. All tests were two-sided, and a *p*-value less than 0.05 was considered statistically significant.

## 3. Results

This study included 111 children who received enoxaparin for non-cerebral artery thrombosis. Their baseline characteristics are summarized in Table 1. The median age at diagnosis was 3 (IQR: 1, 5) months. Most of them (58%) were males with a median body weight of 3.7 (IQR: 2.85, 5.15) kg. The vast majority of them (87%) underwent cardiac catheterization. All cases were diagnosed using ultrasonography. A total of 63% of cases (63%) had complete occlusion, about one-third (32%) had partial occlusion, and 5.4% had complete occlusion on one artery and partial occlusion on the other artery. In total, 31% of the children had femoral thrombosis, 37% had iliac thrombosis, and 32% had both femoral and iliac thrombosis.

The median starting dose of enoxaparin, as shown in Table 2, was 1.37 mg/kg (IQR: 1, 1.5). The median duration of therapy for enoxaparin and anticoagulant therapy was 20 (IQR: 13, 43) and 22 (IQR: 14, 45) days, respectively. The median hemoglobin level was 124 (IQR: 111, 135) g/L at the start of enoxaparin therapy and 122 (114, 132) g/L at the end of it. The median platelet level was 250 (IQR: 172, 338) platelets per mL at the start of therapy and 441 (316, 610) platelets per mL at the end of it. At the end of therapy, 90% of patients achieved complete thrombus resolution, 8.1% achieved partial thrombus resolution, and only 2 patients (1.8%) failed to resolve. The median time to resolution was 18 (14, 37) days. The two patients (1.8%) who did not achieve thrombus resolution had thrombi located in the iliac and femoral sites.

Of 111 patients, only 1 patient (0.9%) had minor bleeding and 2 others (1.8%) had other types of bleeding. No major or fatal bleeding was recorded. Of 110 patients, 85% of patients did not miss any refills. In total, 6 patients (5.5%) missed one refill, 7 patients (6.4%) missed 2 refills, 3 patients (2.7%) missed 3 refills, and only 1 patient missed 4 refills (0.9%). The median level of Anti-factor Xa was 0.52 (IQR: 0.42, 0.58) U/mL. A total of 89% of patients were considered compliant on treatment. Treatment side effects and compliance are demonstrated in Table 3.

**Figure 1:**
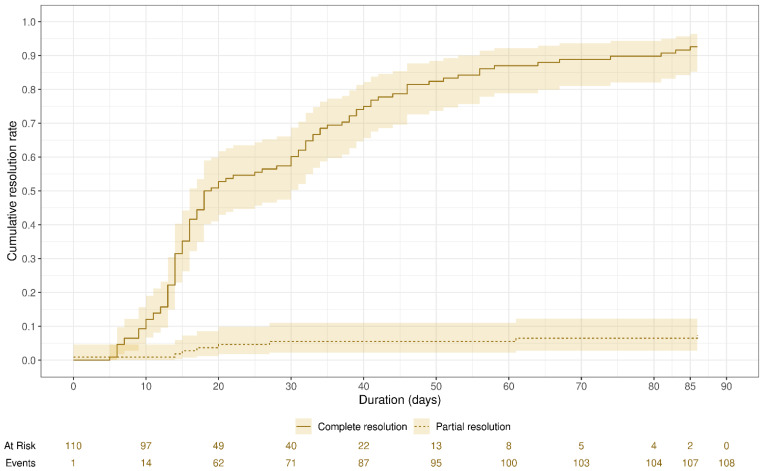
The cumulative resolution rate over time for all patients. Of 110 cases, 108 achieved resolutions (complete resolution = 100, partial resolution = 8). In total, 62/108 (57.4%) achieved resolution after 20 days of therapy, 87/108 (80.5%) achieved resolution after 40 days of therapy, 103/108 (95%) achieved resolution after 70 days of therapy, and 108/108 (100%) achieved resolution after 86 days of therapy.

We performed a multivariate ordinal logistic regression analysis to investigate factors affecting the degree of thrombus resolution (no resolution, partial resolution, and complete resolution; Table 4). The higher the age, the higher the risk of thrombus non-resolution (OR = 1.61 [95% CI: 1.05, 2.46]; *p* = 0.028). The lower the body weight, the higher the risk of thrombus non-resolution (OR = 0.12 [95% CI: 0.02, 0.71]; *p* = 0.019). Patients who had an indwelling catheter were at a greater risk of thrombus non-resolution (OR = 47.2 [95%: 2.95, 75.6); *p* = 0.006). Gender, degree of occlusion, site of lower limb thrombosis, enoxaparin start dose, duration of enoxaparin therapy, duration of anticoagulant therapy, time to resolution, and number of missed refills did not significantly affect the degree of thrombus resolution in the multivariate analysis. Figure 1 represents the cumulative resolution rate over time for all patients.

**Figure 2:**
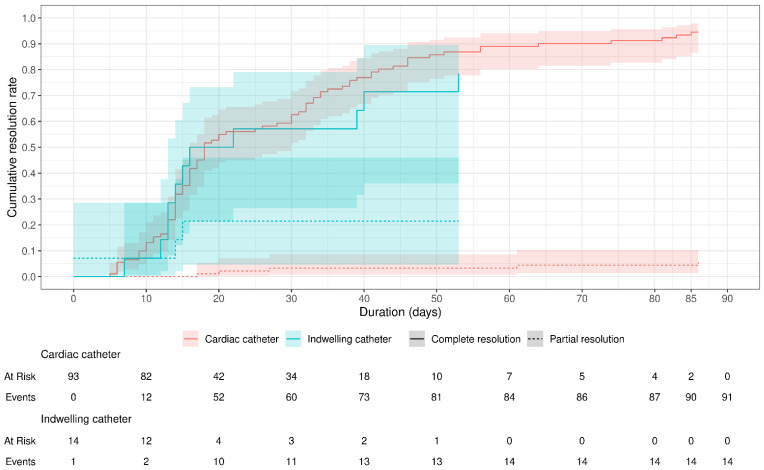
Cumulative resolution rate over time according to the type of catheter. One case was considered as an outlier (time to partial resolution = 303 days) and, hence, was excluded from this analysis. The type of catheterization was unknown for 3 cases, which were also excluded. Of 107 cases, 97 patients achieved complete resolution (86 in the cardiac catheter group and 11 in the indwelling catheter group), 8 patients achieved partial resolution (5 in the cardiac catheter group and 3 in the indwelling group), and 2 patients in the cardiac catheter group failed to resolve.

Figure 2 illustrates the cumulative resolution rate over time according to the type of catheter used. Of 107 cases, 105 achieved resolution and 2 patients who underwent cardiac catheterization did not. In the cardiac catheter group, 52/91 (57.14%) achieved resolution after 20 days of therapy, 73/91 (80.22%) achieved resolution after 40 days of therapy, 86/91 (94.5%) achieved resolution after 70 days of therapy, and 91/91 (100%) achieved resolution after 86 days of therapy. On the other hand, 10/14 (71.43%) patients in the indwelling catheter group achieved resolution after 20 days of therapy, 13/14 (92.86%) achieved resolution after 40 days of therapy, and 14/14 (100%) achieved resolution after 60 days of therapy. So, while a higher proportion of cardiac catheter patients achieved complete resolution (86/91 [91.5%] vs. 11/14 [78.57%]), indwelling catheter patients achieved resolution in a shorter duration of therapy (60 days vs. 86 days).

## 4. Discussion

To the best of our knowledge, this study represents the largest and only retrospective analysis of pediatric patients with catheter-related arterial thrombosis (CAT) in the Middle East reported in the literature to date. We aimed to evaluate the effectiveness of enoxaparin in treating pediatric arterial thrombosis, specifically CAT, at a single center in Saudi Arabia. Our findings show that enoxaparin is highly effective, with a 90% complete thrombus resolution rate, 8.1% partial resolution, and only 1.8% non-resolution. In comparison, a recent study of 101 patients with a median age of 2.2 months found that 39.6% had congenital heart disease and 22.8% had infections. Symptomatic thrombosis in an extremity was common, with 78% being catheter-associated. Anticoagulation therapy, given to 81% for a median of 35 days, resulted in 70% achieving complete resolution after 90 days. Infection-associated thrombosis was linked to long-term complications in 18 patients, with a bleeding rate of 11% [9]. Also, compared to our study, which demonstrated a 90% complete resolution rate of CAT using enoxaparin, another study analyzing 242 CAT cases treated primarily with heparin alone showed a 71.5% complete resolution rate. In particular, transitioning from heparin to acetylsalicylic acid (ASA) in cases with partial or no resolution did not improve the resolution rate, highlighting the superior effectiveness of enoxaparin in achieving higher resolution outcomes [6]. These findings reinforce the effectiveness of enoxaparin in treating pediatric CAT and highlight the importance of tailored anticoagulation therapy in achieving favorable outcomes. One of the significant insights from our study is the association between higher age and lower body weight with a higher risk of non-resolution, while in another study, no definitive predictors of complete resolution were identified [9]. Another study found that factors associated with complete thrombus resolution included a shorter time from intervention to the diagnosis of catheter-related arterial thrombosis, as well as the involvement of the iliac and/or femoral arteries [7]. Additionally, in our study, patients with indwelling catheters had a greater risk of non-resolution compared to those with cardiac catheters, indicating that the type of catheter plays a critical role in treatment outcomes. The median duration of enoxaparin therapy was 20 days, which aligns with the current studies but highlights the need for more precise guidelines. Previous studies indicate that extending the duration of anticoagulant therapy improves complete response (CR) rates. For instance, Crameri et al. reported a 66% CR rate in their cohort within the first six weeks of treatment [6]. Cohen et al. reported a 60.7% complete response (CR) rate after 28 days of anticoagulation therapy [7]. Furthermore, Glatz et al. observed an 89% complete response (CR) rate in patients following 12 weeks of therapy [10]. In another study, 70% of patients achieved complete resolution after 90 days of anticoagulation. However, a significant number of patients developed long-term sequelae due to arterial insufficiency. Those diagnosed with AT who had sepsis or infection were more likely to experience long-term complications [9]. These findings challenge the ACCP Guidelines, which recommend a short course of anticoagulation (5–7 days) [11]. The variability in treatment duration underscores the necessity for individualized treatment plans based on patient response and the specifics of each case. Our study also showed that enoxaparin is well tolerated among pediatric patients, with minimal side effects. Only a small number of patients experienced minor bleeding, and no major or fatal bleeding events were recorded. This supports the preference for enoxaparin due to its predictable pharmacokinetics, ease of administration, and lower risk of adverse effects [11]. The high compliance rate observed in our study is encouraging, with 89% of patients adhering to the treatment regimen. This compliance is crucial for the success of anticoagulant therapy and highlights the importance of patient and caregiver education regarding the importance of adherence to prescribed treatments. While our study provides valuable insights, it is not without limitations. The retrospective nature of the study and the single-center setting may limit the generalizability of the findings. Additionally, the lack of a control group means we cannot definitively compare the effectiveness of enoxaparin with other anticoagulants or treatment modalities. Our study demonstrates that enoxaparin is an effective and safe treatment for catheter-related arterial thrombosis in pediatric patients. The high rate of thrombus resolution and minimal side effects make it a viable option for managing this condition. However, further research is needed to refine treatment durations and address specific risk factors, ultimately guiding clinicians in providing the best care for their pediatric patients.

## 5. Conclusions

Enoxaparin proves to be highly effective and safe for treating catheter-related arterial thrombosis in pediatric patients, achieving a 90% complete resolution rate with minimal side effects. Key factors affecting outcomes include patient age, weight, and catheter type, emphasizing the need for personalized treatment plans. High compliance rates support enoxaparin’s reliability, but further research is needed to refine treatment durations and improve guidelines for pediatric care.

## Figures and Tables

**Table 1:** Baseline characteristics.

Characteristic	Value ^1^	N
Age at diagnosis (months)	3.0 (1.0, 5.0)	111
Gender		111
Male	64 (58%)	
Female	47 (42%)	
Body weight (kg)	3.70 (2.85, 5.15)	111
Catheter type		108
Cardiac catheter	94 (87%)	
Indwelling catheter	14 (13%)	
Type of radiological test		111
US	111 (100%)	
Degree of occlusion		111
Bilateral complete	70 (63%)	
Bilateral partial	35 (32%)	
Partial and Complete	6 (5.4%)	
Lower limb thrombosis		111
Femoral	34 (31%)	
Iliac	41 (37%)	
Both	36 (32%)	

^1^ Median (IQR); *n* (%).

**Table 2:** Treatment characteristics and outcomes.

Characteristic	Value ^1^	N
Enoxaparin start dose (mg/kg)	1.37 (1.00, 1.50)	111
Duration of enoxaparin therapy (days)	20 (13, 43)	111
Duration of anticoagulant (days)	22 (14, 45)	111
Hemoglobin level at the start of enoxaparin therapy (g/L)	124 (111, 135)	110
Hemoglobin level at the end of enoxaparin therapy (g/L)	122 (114, 132)	68
Platelet level at the start of enoxaparin therapy (per mL)	250 (172, 338)	110
Platelet level at the end of enoxaparin therapy (per mL)	441 (316, 610)	67
Resolution degree		111
No resolution	2 (1.8%)	
Partial resolution	9 (8.1%)	
Complete resolution	100 (90%)	
Time to resolution (days)	18 (14, 37)	109

^1^ Median (IQR); *n* (%).

**Table 3:** Treatment side effects and compliance.

Characteristic	Value ^1^	N
Bleeding		111
No bleeding	108 (97%)	
Minor bleeding	1 (0.9%)	
Major bleeding	0 (0%)	
Fatal bleeding	0 (0%)	
Other types of bleeding	2 (1.8%)	
Number of missed refills		110
0	93 (85%)	
1	6 (5.5%)	
2	7 (6.4%)	
3	3 (2.7%)	
4	1 (0.9%)	
Anti-factor Xa level (U/mL)	0.52 (0.42, 0.58)	108
Compliance *	95 (89%)	107

^1^ *n* (%); median (IQR); * non-compliance was considered whenever Anti-factor Xa level was <0.5 U/mL and ≥1 refills were missed.

**Table 4:** Multivariate analysis for factors affecting thrombus resolution.

Characteristic	Odds Ratio (OR)	95% CI ^1^	*p*-Value
Age (months)	1.61	1.05, 2.46	0.028
Gender			
Female	-	-	
Male	2.89	0.31, 26.8	0.4
Body weight (kg)	0.12	0.02, 0.71	0.019
Catheter			
Cardiac catheter	-	-	
Indwelling catheter	47.2	2.95, 75.6	0.006
Degree of occlusion			
Bilateral complete	-	-	
Bilateral partial	0.45	0.03, 6.13	0.6
Partial and complete	0.08	0.00, 79.0	0.5
Lower limb thrombosis			
Femoral	-	-	
Iliac	6.46	0.41, 103	0.2
Both	1.00	0.05, 19.8	>0.9
Enoxaparin start dose	0.78	0.02, 28.1	0.9
Duration of enoxaparin therapy (days)	0.98	0.83, 1.17	0.8
Duration of anticoagulant (days)	1.04	0.88, 1.24	0.6
Time to resolution (days)	1.00	0.97, 1.04	>0.9
Number of missed refills	1.39	0.35, 5.57	0.6

^1^ CI = confidence interval.

## Data Availability

The data that support the findings of this study are available from King Abdullah International Medical Research Center, but restrictions apply to the availability of these data, which were used under license for the current study, and so are not publicly available. Data are, however, available from the authors upon reasonable request and with permission of King Abdullah International Medical Research Center.
